# The complete chloroplast genome of Chinese medicinal herb *Belamcanda chinensis* (L.) Redouté (Iridaceae)

**DOI:** 10.1080/23802359.2020.1866455

**Published:** 2021-02-08

**Authors:** Cuicui Li, Saiwen Hu, Yining Ding, Guangyao Bi, Chun Su, Zhi Xia

**Affiliations:** a College of Agronomy, Henan Agricultural University, Zhengzhou, Henan, China; b College of Life Sciences, Northwest A&F University, Yangling, Shanxi, China

**Keywords:** *Belamcanda chinensis*, chloroplast genome, medicine herb, phylogenetics, Iridaceae

## Abstract

The species of *Belamcanda chinensis* (L.) Redouté. is one of the Chinese traditional medicinal herb. In this study, we first report the complete chloroplast (cp) genome of *B. chinensis*. The chloroplast (cp) genome was determined to be 153,735 bp and the GC contents was 37.9%. The sequence includes a large single-copy (LSC) region of 83, 199 bp, a small single-copy (SSC) region of 18,168 bp, and two separated inverted regions of 26,184 bp each. It contains 132 genes, including 86 protein-coding genes, 38 tRNA genes, and 8 rRNA genes. Based on 10 chloroplast genomes data, the maximum likelihood phylogenetic analysis revealed that *B. chinensis* was sister to *Iris* (Bootstrap = 100%) within Iridaceae. This result will be helpful for the conservation and breeding programs of the *B. chinensis.*

*Belamcanda chinensis* (L.) Redouté. is a large perennial herb belonging to the family Iridaceae. It is an important species in traditional Chinese medicine and is widely distributed in China and Southeast Asia. The rhizome of *B. chinensis* has been used as a traditional medicine for the treatment of inflammation, asthma, tonsillitis, and many other throat disorders (Lee et al. 2015). In this study, we first report the complete chloroplast genome (cp genome) of traditional Chinese medicinal herb *B. chinensis*, and inferred phylogenetic relationships of this species as well as the genus *Belamcanda* to other taxa in Iridaceae.

The fresh and healthy leaves of Chinese medicine species *B. chinensis* was collected from Nanzhao County, Nanyang City, Henan Province, China (33°25′42″N, 112°25′54″E). The voucher specimen (voucher no.: XZ-2020-10) was deposited at the herbarium of Henan Agricultural University (HEAC). The total genomic DNA was extracted following the CTAB method (Doyle and Doyle 1987). After DNA extraction, 1 µg genomic DNA was randomly fragmented by Covaris, followed by fragment selection by Agencourt AMPure XP-Medium kit to an average size of 200–400 bp. Then the genomic library (paired-end, PE 150 bp) was sequenced on an Illumina Hiseq 4000 platform at Beijing Genomics Institute (Shenzhen, China). Totally 1.63 Gb sequence reads were obtained and used to assemble the chloroplast genome after filtering and trimming the low-quality reads and adaptor sequences. Then, NOVOPlasty (Dierckxsens et al. 2017) was used *de novo* to assemble the chloroplast genome with manual adjustment and annotation using Geneious version 11.0.3 (Kearse et al. 2012). *Iris sanguinea* (GenBank accession number: KT626943) was used as reference plastid genome for assembling and annotation. The tRNA genes were annotated on ARAGORN (Laslett and Canback 2004).

The complete chloroplast genome of traditional Chinese medicine herb *B. chinensis* (GenBank accession number: MW039136) has a length of 153,735 bp with a typical circle structure and a GC content of 37.9%. It consists of a large single-copy region (LSC, 83,199 bp, 36.0% GC content), a small single-copy region (SSC, 18,168 bp, 31.5% GC content), and two inverted repeat regions (IR, 26,184 bp, 43.1% GC content). The genome harbors 132 genes, including 86 protein-coding genes, 38 tRNA genes, and 8 rRNA genes. Among these, 15 genes (*trn*K-UUU, *rps*16, *trn*G-UCC, *atp*F, *rpo*C1, *trn*L-UAA, *trn*V-UAC, *pet*B, *pet*D, *rpl*16, *rpl*2, *ndh*B, *trn*I-GUA, *trn*A-UGC, and *ndh*A) had one intron, while three genes (*rps*12, *clp*P, and *ycf*3) contained two introns.

A total of 9 complete chloroplast genomes of *B. chinensis* together with its related species were utilized to clarify the phylogenetic position of *B. chinensis*, using *Lycoris sanguinea* Maxim. and *Lycoris aurea* (L’Héritier) Herbert as the outgroups. The sequence alignment was conducted with MAFFT v7.3 (Katoh and Standley 2013). A maximum likelihood analysis was performed with the RAxML software (Stamatakis 2014) using 1000 bootstrap replicates. These analyses used the GTR substitution model with gamma-distributed rate heterogeneity among sites and the proportion of invariable sites estimated from the data. The phylogenetic tree indicates that all sampled species within Iridaceae formed one monophyletic clade with BS = 100%. Four species of *Iris* formed one monophyletic clade with BS = 100%, and *B. chinensis* is sister to the genus *Iris* with BS = 100% (Figure 1). The chloroplast genomes resource may be utilized for DNA barcoding, conservation genetics, and breeding of cultivar *B. chinensis* in the future.

**Figure 1.**
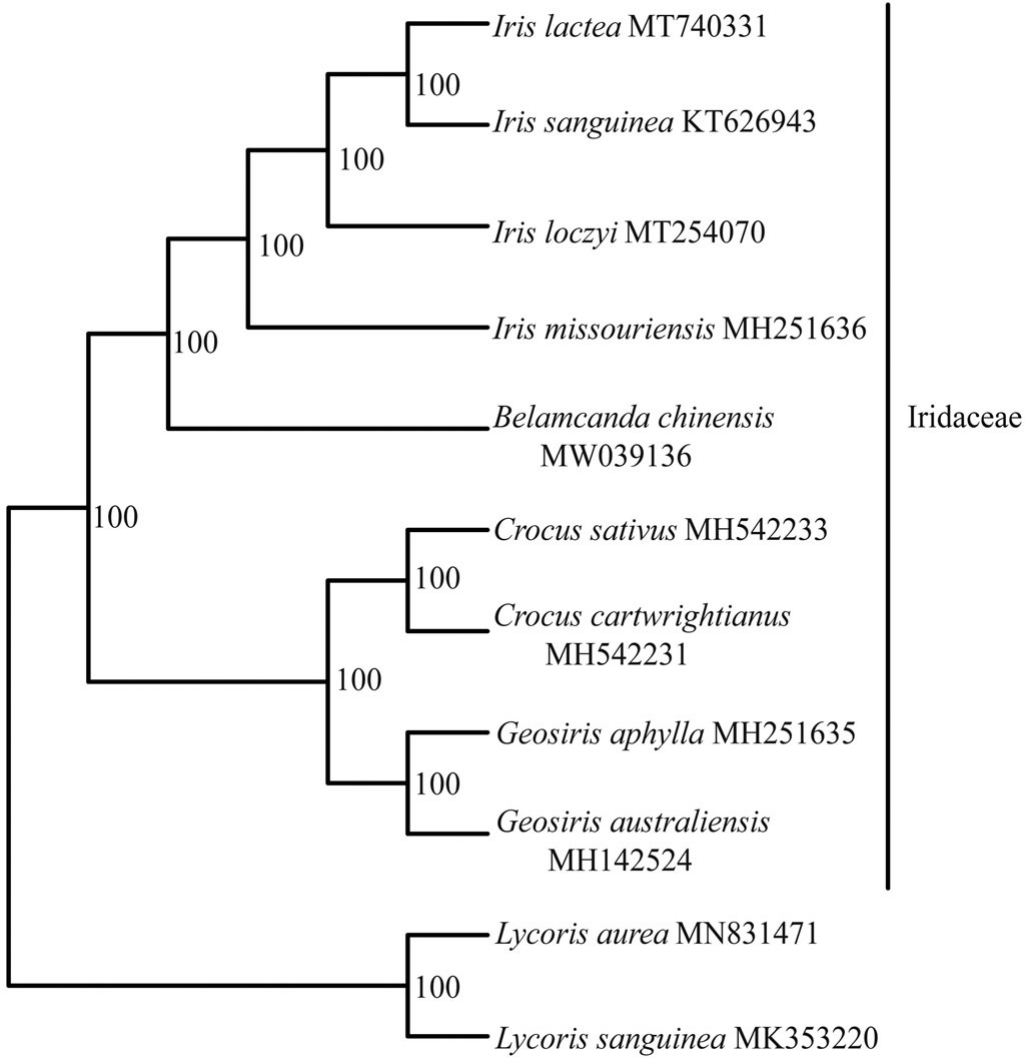
Maximum likelihood phylogenetic tree based on 9 complete chloroplast genome sequences. The number on each node indicates the bootstrap value.

## Data Availability

The complete chloroplast genome sequence of *Belamcanda chinensis* has been submitted to the GenBank (https://www.ncbi.nlm.nih.gov/genbank/), and the accession number is MW039136. This sequence will be released immediately after process by the NCBI staff. Then, the data that support the findings of this study is openly available in GenBank.
